# Sensitive and Accurate Proteome Profiling of Embryogenesis Using Real-Time Search and TMTproC Quantification

**DOI:** 10.1016/j.mcpro.2024.100899

**Published:** 2024-12-24

**Authors:** Alex N.T. Johnson, Jingjing Huang, Argit Marishta, Edward R. Cruz, Andrea Mariossi, William D. Barshop, Jesse D. Canterbury, Rafael Melani, David Bergen, Vlad Zabrouskov, Michael S. Levine, Eric Wieschaus, Graeme C. McAlister, Martin Wühr

**Affiliations:** 1 Department of Chemical and Biological Engineering, Princeton University, Princeton, New Jersey, United States; 2 Lewis-Sigler Institute for Integrative Genomics, Princeton University, Princeton, New Jersey, United States; 3 Thermo Fisher Scientific, San Jose, California, United States; 4 Department of Molecular Biology, Princeton University, Princeton, New Jersey, United States

**Keywords:** multiplexing, TMTpro, complementary ion quantification, *Drosophila melanogaster*, *Xenopus laevis*, *Ciona robust*a

## Abstract

Multiplexed proteomics has become a powerful tool for investigating biological systems. Using balancer–peptide conjugates (*e.g.*, TMTproC complementary ions) in the MS2 spectra for quantification circumvents the ratio distortion problem inherent in multiplexed proteomics. However, TMTproC quantification scans require long Orbitrap transients and extended ion injection times to achieve sufficient ion statistics and spectral resolution. Real-time search (RTS) algorithms have demonstrated increased speed and sensitivity by selectively informing precursor peak quantification. Here, we combine complementary ion quantification with RTS (TMTproC-RTS) to enhance sensitivity while maintaining accuracy and precision in quantitative proteomics at the MS2 level. We demonstrate the utility of this method by quantifying protein dynamics during the embryonic development of *Drosophila melanogaster* (fly), *Ciona robusta* (sea squirt), and *Xenopus laevis* (frog). We quantify 7.8k, 8.6k, and 12.7k proteins in each organism, which is an improvement of 12%, 13%, and 14%, respectively, compared with naive TMTproC analysis. For all three organisms, the newly acquired data outperform previously published datasets and provide a diverse, deep, and accurate database of protein dynamics during embryogenesis, which will advance the study of evolutionary comparison in early embryogenesis.

## Highlights

• Combined complementary ion quantification and real-time search (TMTproC-RTS).
• Boosted sensitivity in MS2-based multiplexed proteomics.
• Quantified protein dynamics in three model organisms during embryogenesis.
• Achieved 12 to 14% more protein identifications compared with TMTproC methods.

## Graphical Abstract

**Figure:**
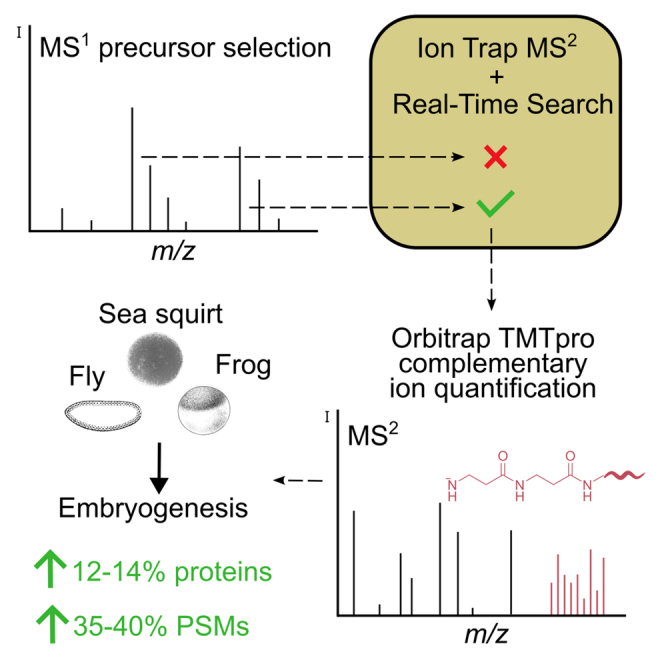

## In Brief

This study introduces TMTproC-RTS, a method combining complementary ion quantification and real-time search, to enhance sensitivity without compromising accuracy in MS2-based quantitative proteomics. Applied to embryogenesis in *Drosophila melanogaster*, *Ciona robusta*, and *Xenopus laevis*, the method quantified up to 14% more proteins than prior approaches, creating the most comprehensive dataset of protein dynamics for these species to date, enabling evolutionary comparisons and advancing developmental biology research.

Over the past 2 decades, quantitative multiplexed proteomics has advanced rapidly, deepening our understanding of a range of biological systems. Among these advancements, isobaric tagging stands out as a key contributor, addressing challenges inherent in label-free proteomics (1, 2). Isobaric tags (*e.g.*, tandem mass tags, TMT and TMTpro) encode information about sample identity (3). Peptides from multiple samples are labeled with one of several isobaric tags and combined before analysis in a single run. The different quantification channels are encoded by the distribution of heavy isotopes between the reporter and balancer region of the tags. Multiplexing with TMTpro allows for the quantification of proteins from up to 18 samples in a single experiment, increasing sample throughput and quantitative reproducibility (3).

Despite these advancements, challenges persist, most notably quantification inaccuracy caused by interference from coeluting peptides. Peptides with similar mass-to-charge ratios that elute at the same time as a peptide of interest are coisolated and cofragmented. Reporter ions cleaved from TMT(pro) tags on interfering peptides are indistinguishable from those originating from the peptide of interest. The resulting quantification is typically significantly distorted (4). Numerous approaches to solve interference have been implemented, such as mass spectrometry (MS3)-based methods that use an additional gas phase purification (5, 6), ion–ion reactions (7), or ion mobility (8, 9), to reduce sample complexity and post hoc computational corrections (10, 11).

Using the balancer–peptide conjugates (complementary ions) in the MS2 spectra for quantification circumvents the ratio distortion problem (12). After precursor fragmentation, reporter ions are cleaved from TMT(pro) tags, leaving the balancer region of the tag bound to the peptide of interest. Because peptide-level *m/z* differences are preserved, complementary ions from the peptide of interest can be distinguished from interfering peptides. Complementary ion quantification of TMTpro-tagged samples (TMTproC) is more accurate than MS2 or MS3 reporter ion–based quantification (13). Furthermore, TMTproC quantification is done on the MS2 level, avoiding the need for additional MS3 scans, improving the spectral acquisition rate and sensitivity.

TMTproC has several drawbacks compared with reporter ion quantification. First, because complementary ions are found at higher *m/z* ratios, small differences in mass defects between ^13^C and ^15^N cannot be resolved with typical Orbitrap transient lengths (14). The number of samples that can be analyzed with TMTpro is therefore reduced from 18 to 9 channels. Second, complementary ions form less efficiently than reporter ions, so more precursor ions must be isolated for accurate quantification. TMTproC scans therefore require longer ion injection times (IITs) for sufficient ion statistics (13).

Real-time search (RTS) algorithms have been shown to increase sensitivity of SPS-MS3 methods by informed selection of precursor peaks for quantification (15). Each precursor is fragmented, and a fast and low-resolution MS2 scan is collected with an ion trap. The spectrum is then searched against an appropriate FASTA file. If the spectra can be matched to a peptide, an MS3 scan is collected. This strategy improves duty cycles by eliminating MS3 scans for precursors that do not match a peptide, affording more time to quantifiable peptides.

Here, we combine complementary ion quantification with RTS (TMTproC-RTS) to improve sensitivity while maintaining accuracy and precision in quantitative proteomics experiments. Exploratory MS2 scans are collected in the ion trap and searched in real time against a FASTA file. If a peptide match is found, a second MS2 scan is collected in the high-resolution Orbitrap with longer IIT. Similar to MS3-RTS, the method improves instrument duty cycle by eliminating lengthy TMTproC MS2 scans of precursors without a peptide match. We benchmark the sensitivity gained from this method by quantifying proteome dynamics throughout the development of *Drosophila melanogaster*, *Ciona robusta*, and *Xenopus laevis* embryos. For each of these organisms, we outperform the previously published comparable datasets, thereby providing a diverse, deep, and accurate database of protein dynamics during embryogenesis.

## Experimental procedures

### Sample Preparation

Peptide mixtures from five cell types were prepared for use in this study: a two-organism interference model (HeLa, yeast) and time courses of embryonic development in *D. melanogaster*, *C. robusta*, and *X. laevis*. Peptides from the Pierce TMT11plex Yeast Digest Standard (Thermo Scientific; A40939) were used without modification.

The lysis protocols differed by cell type. Briefly, *D. melanogaster* embryos, *C. robusta* embryos, and HeLa cells were resuspended in 50 mM Hepes, pH 7.2 (Sigma; H3375), 2% SDS (Thermo Fisher; AM9820), and Pierce protease inhibitor (1 tablet per 10 ml; Thermo Fisher; PI88666). Cells/embryos were lysed by direct tip sonication for five cycles of 30 s at 50% amplitude and 15 s on ice between cycles. Yeast cells were lysed by cryomilling and later resuspended in the same lysis buffer. After sonication/cryomilling, cell debris was pelleted at 4 kg for 15 min, and the sample preparation was continued with supernatant.

*X. laevis* embryos were lysed as previously described (16). Briefly, a lysis buffer consisting of 250 mM sucrose, 1% Nonidet P-40, 10 mM EDTA, 25 mM Hepes (pH = 7.2), 10 μM cytochalasin D, and Pierce protease inhibitor (1 tablet per 10 ml) was added to the embryos and pipetted up and down 15 times. Samples were then incubated on ice for 10 min and sonicated for 10 s. Yolk was then removed with a soft spin at 2500 rcf for 4 min at 4 °C. Next, 100 mM Hepes, pH 7.2, and 2% SDS were then added to denature proteins.

Sample preparation for all samples was identical from here forward as previously described (17). Briefly, 5 mM DTT (Thermo Fisher; 16568-0050) was added to lysates and heated at 60 °C for 20 min. After cooling to room temperature, lysates were reacted with 20 mM *N*-ethylmaleimide (Thermo Fisher; 23030) at room temperature for 20 min. DTT was then added to a concentration of 10 mM and incubated at room temperature for 10 min.

A 1:1 mixture of Sera-Mag Speedbead carboxylate-modified magnetic beads (Cytiva; 45152105050250 and 65152105050250) was then added to samples at a bead:protein ratio of 15:1. Ethanol was added to samples to a concentration of 50% and mixed at 1400 RPM for 5 min on a thermomixer to initiate protein binding. Magnets were used to bind beads to the side of the tube, and the supernatant was discarded. Beads were washed three times with 80% ethanol before resuspension in 2 M guanidinium hydrochloride (Sigma, 369079), 10 mM EPPS (pH 8.5) (Sigma; E9502), and 20 ng μL^−1^ of LysC (Wako; 125-05061). Samples were digested overnight at room temperature while mixing. Samples were then diluted with 10 mM EPPS (pH 8.5) to a guanidinium hydrochloride concentration of 0.5 M. Trypsin (Promega; V5111) and LysC were added to a concentration of 10 ng μL^−1^ and 20 ng μL^−1^, respectively, and samples were digested overnight at 37 °C while mixing. Magnetic beads were then removed from the sample, and the supernatant was vacuum dried at room temperature. Samples were then resuspended in 200 mM EPPS (pH 8.0) to concentration of 1 μg μL^−1^. TMTpro (Thermo Fisher; A52045) was added at a mass ratio of 5:1 tag:peptide and allowed to react for 2 h at room temperature. For the two-organism interference sample, TMTpro tags were premixed at the defined ratios before peptide labeling. The reaction was quenched with 1% hydroxylamine (Sigma; 467804) for 30 min at room temperature. Samples from all channels were combined into one tube before acidification with 5% phosphoric acid. Combined samples were then ultracentrifuged at 100,000*g* at 4 °C for an hour to pellet undigested proteins. The supernatants were then vacuum-dried at room temperature to remove acetonitrile. The HeLa–yeast peptides were resuspended in HPLC-grade water, and stage-tipping was performed to desalt the samples (18). Samples were resuspended to 0.5 μg μL^−1^ in 1% formic acid (FA) and HPLC-grade water before analysis.

Samples from embryonic development were resuspended after ultracentrifugation in 10 mM ammonium bicarbonate (pH 8.0), sonicated, and then fractionated by medium pH reverse-phase HPLC (Zorbax 300Extend C18, 4.6 × 250 mm column; Agilent) with 10 mM ammonium bicarbonate, pH 8.0, using an acetonitrile gradient from 5% to 30%. Fractions were collected into a 96-well plate. These fractions were pooled into 24 fractions by alternating the wells in the plate (19). Each fraction was dried and resuspended in 30 μl of HPLC water and acidified to pH <2 with phosphoric acid. Stage-tipping was performed to desalt the samples (18). Samples were resuspended to 0.5 μg μL^−1^ in 1% FA and HPLC-grade water before analysis.

### *D. melanogaster* Embryo Collection

Canton special *D. melanogaster* flies were placed in collection chambers with apple juice–agar medium Petri dish plates. Plates were changed every 2 to 4 h and either heat-fixed immediately or incubated first at room temperature for 2 to 8 h and then heat-fixed. For heat-fixation, 5 to 10 ml 5% bleach was added to the plate and incubated for 1 to 2 min. Then, the embryos were swirled around with a brush and poured in a 2 cm (diameter) mesh metal basket, which catches the embryos and discards the bleach. While in the mesh, embryos were then rinsed with running water for 10 to 20 s and dumped in a vial with 5 ml boiling Triton salt solution (0.4% NaCl in 0.01% Triton X-100). Immediately, the vial was transferred in an ice bucket, and 1 ml ice-cold water was added. After 5 min, the mesh basked was removed with tweezers, and the Triton salt solution was pipetted out, leaving only the embryos in the bottom of the vial. A mixture of 5 ml heptane and 5 ml methanol was then added to the vial and vortexed for 20 s. The vial rested until the two liquid phases separated, and the embryos fell to the bottom of the vial. The embryos at the bottom of the vial were then pipetted out, collected in an Eppendorf tube, and rinsed three times with methanol. The embryos were then either stored in the freezer or sorted and staged manually in a brightfield stereo microscope (20).

### *C. robusta* Embryo Collection

Adult *C. robusta*, previously known as *Ciona intestinalis* type A (21), were collected by M-Rep in San Diego, CA. Adults were kept under continuous illumination to stimulate gamete production. Embryos were fertilized accordingly (22) and incubated at 18 °C. Nine different stages spanning important milestones of embryogenesis including fertilization, maternal–zygotic transition, gastrulation, neurulation, tailbud formation, and swimming larvae were selected and collected following the staging nomenclature in the study by Hotta *et al*. (23). Around 3000 synchronized embryos were flash-frozen in liquid nitrogen for each stage. Once all stages were collected, sample preparation proceeded as detailed previously.

### *X. laevis* Embryo Collection

Mature *X. laevis* females and males were purchased from Xenopus1 and maintained by Laboratory Animal Resources at the Princeton University. All animal procedures are approved under Institutional Animal Care and Use Committee protocol 2070. Unfertilized eggs and male testes were collected following standard laboratory procedures previously described (24). For testes collection, *X. laevis* males are euthanized in 0.1% (w/v) tricaine methanesulfonate (MS-222; Syndel’s Syncaine) and then sacrificed by pithing. The testes are isolated and stored at 4 °C in oocyte culture medium that was exchanged daily for up to 1 week (1 l: 13.7 g Leibovitz’s L-15 Medium Powder [ThermoFisher Scientific; #41300039], 8.3 ml penicillin–streptomycin [ThermoFisher Scientific; #15140122], 0.67 g bovine serum albumin). For egg collection, female frogs were injected with 500 U of human chorionic gonadotropin (CG10; Sigma) and kept at 16 °C in Marc’s modified Ringer’s solution for 16 h before collection (1X MMR: 5 mM Hepes [pH 7.8], 0.1 mM EDTA, 100 mM NaCl, 2 mM KCl, 1 mM MgCl_2_, and 2 mM CaCl_2_). For *in vitro* fertilization, female eggs collected in 1X MMR buffer are cleaned, and preactivated eggs are removed. Half of one male testis was used per 500 eggs by crushing in 1X MMR buffer with a sterile pestle and then mixing with the unfertilized eggs. The mixture was incubated at 16 °C for 5 min, followed by mixing and an additional 5-min incubation. Fertilization was induced by flooding the eggs with 0.1X MMR. After 1 h at 16 °C, embryo jelly coats were removed by incubating with 2% cysteine in 0.1X MMR for 5 min, and the embryos were thoroughly washed with 0.1X MMR to remove residual cysteine. Embryos were grown and staged by Nieuwkoop and Faber nomenclature (25) at 16 °C and then flash frozen at desired time points. Embryo lysis and preparation for MS analysis was performed as described previously.

### MS Methods

Samples were analyzed on a Vanquish Neo UHPLC System coupled to an Orbitrap Ascend Tribrid mass spectrometer (ThermoFisher Scientific). Peptides were separated on an Ionopticks Aurora Series emitter column (25 cm × 75 μm ID, 1.6 μm C18) held at 60 °C during separation by an in-house built column oven. Solvent A consisted of 0.5% dimethyl sulfoxide (LC–MS grade; Life Technologies), 0.1% FA (98%+; TCI America) in water (LC–MS grade; OmniSolv, VWR), and solvent B consisted of 0.5% dimethyl sulfoxide and 0.1% FA in acetonitrile (LC–MS grade; OmniSolv, MilliporeSigma).

The mass spectrometer was set to analyze positively charged ions in a data-dependent MS2 mode, recording centroid data with the RF lens level at 60%. Full scans were taken with the Orbitrap at 120k resolution with an automatic gain control (AGC) target of 4E5 charges, maximum IIT of 50 ms, and scan range of 350 to 1400 *m/z* with wide quadrupole isolation enabled. Maximum cycle time between MS1 scans was set to 3 s.

Following the survey scan, the following filters were applied for triggering MS2 scans. Monoisotopic peak selection was enabled (“Peptide Mode”). Isolated masses were excluded for 60 s after triggering with a mass tolerance window of ±10 ppm, while also excluding isotopes and different charge states of the isolated species. Ions with z = 2+ and 3+ were analyzed if their *m/z* ratio was between 500 and 1074 (z = 2+) or 350 to 1381 (z = 3+) to ensure visibility of the complementary ion clusters in a normal range MS2 scan.

The following settings were used for exploratory ion trap MS2 scans. AGC target was set to 3E4 charges, and the maximum IIT was 13 ms. The quadrupole was utilized for isolation with an isolation width of 0.5 Da, and ions were fragmented with higher-energy collision dissociation (HCD) at a normalized collision energy of 35%. The scan rate was set to turbo over a range of 200 to 1400 *m/z*.

Exploratory ion trap scans were searched against an appropriate FASTA file with common contaminants. Variable methionine oxidation, static TMTpro modifications on lysines/peptide N termini, and static *N*-ethylmaleimide modification of cysteine residues were allowed. The maximum variable modifications was set to 2 and the maximum missed cleavages at 1. RTS score thresholds were set as follows. Peptide were deemed acceptable if their cross-correlation was greater than 1.4, their delta cross-correlation was greater than or equal to 0.2, and their absolute precursor parts per million deviation was less than or equal to 10. The “use as trigger only” feature was enabled. For fractionated samples, false discovery rate (FDR) filtering was enabled with a threshold of 10%. For runs where a subset of the proteome was desired, an exclusion tag was added to the header of proteins in the FASTA file that were undesired.

Following a successful exploratory scan, unless stated otherwise in the *Results* section, the following settings were used for MS2 TMTproC scans in the Orbitrap. The AGC target was set to 7.5E4 charges, and the maximum IITs was 91 ms for unfractionated samples and 123 ms for fractionated samples. The quadrupole utilized an isolation width of 0.4 Da, and ions were fragmented with collision-induced dissociation (CID) at a normalized collision energy of 30% and an activation time of 10 ms. An Orbitrap resolution of 45k was used for unfractionated samples and a resolution of 60k for fractionated samples.

### Data Analysis

The data were analyzed using the Gygi Lab GFY software licensed from Harvard. Incorrectly assigned precursor charge state as well as incorrectly determined monoisotopic peaks were corrected (15). Assignment of MS2 spectra was performed using the Comet algorithm, version 3.12 by searching the data against an appropriate FASTA file along with common contaminants. Human and yeast FASTA files were acquired from UniProt on July 8, 2016 (SwissProt + Trembl). For *X. laevis*, the Xenbase (http://www.xenbase.org/, Research Resource Identifier: SCR_003280) genome and gene models v10.1 were used (26). For *D. melanogaster*, the Flybase genome and gene models version FB2016_05 were used (27). For *C. robusta*, the KY21 genome and gene model were used (28). Database sizes for each search were as follows: yeast (6,163 entries), human and yeast concatenated (77,448 entries), *D. melanogaster* (30,599 entries), *C. robusta* (27,752 entries), and *X. laevis* (34,682 entries).

Comet searches were performed using a 20 ppm precursor ion tolerance with the requirement that both N- and C-terminal peptide ends are consistent with the protease specificities of LysC and Trypsin. Only TMTproC scans performed in the Orbitrap were searched by specifying CID collision energy in the Comet parameter file. The fragment ion tolerance of the MS2 spectrum was set to 0.02 Da. TMTpro (+304.2071 Da) was set as a static modification on N termini and lysine residues, and *N*-ethyl maleimide (+125.047679 Da) was set as a static modification on cysteine residues. Oxidation of methionine (+15.99492 Da) was set as a variable modification. The maximum variable modifications were set to 2, and maximum missed cleavages were set to 1. The target-decoy strategy was used to construct a second database of reversed sequences that were used to estimate the FDR on the peptide level (29). A peptide spectral match (PSM)–level FDR of 1% was obtained by applying the target decoy strategy with linear discriminant analysis as described previously (30). Peptides were assigned to proteins, and a second filtering step to obtain a 1% FDR on the protein level was applied. Peptides that matched multiple proteins were assigned to the proteins with the most unique peptides (31).

The complement reporter ion cluster *m/z* was calculated from theoretical TMTpro fragmentation losses, and the observed intensities were extracted. All downstream analyses were performed using R Statistical Software (version 4.3.1; R Core Team 2021). TMTpro isotopic impurity corrections were done with a custom R script (available at: https://github.com/wuhrlab). Measured isotopic impurities for the complement and reporter regions of each TMTpro tag (13) as well as theoretical peptide isotopic distributions were used to determine the proportion of TMTpro impurities expected in the isolated peak for each tag. This impurity matrix was used to solve an overdetermined system of equations Ax = B with QR decomposition to determine the correct complementary ion ratios.

Proteins from each organism were mapped to the human proteome using OrthoFinder, version 3.0 (32). FASTA files were first reduced so that each gene was represented by its longest isoform before mapping. About 15,754 total orthogroups were generated. Many-to-many relationships were allowed between all organisms, and when multiple proteins from an orthogroup were detected in an organism, the protein with the most quantified peptides was selected.

### Experimental Design and Statistical Rationale

Comparisons between TMTproC and TMTproC-RTS on target protein lists were performed in technical triplicate (n = 3) and plotted with standard deviations. Time courses of embryogenesis were analyzed in technical duplicate (n = 2) and were highly reproducible. Peptides with a total signal:Fourier transform noise of at least 90 (or when using less than a 9-plex, 10 times the number of labeled channels) were considered quantified. Peptide quantification was normalized so that peptides stemming from mitochondrial proteins were constant across each time course. Aggregation of peptide-level quantification into protein quantification for the embryo time series was done by taking the median-corrected signal for all peptides mapped to each protein.

Hierarchical k-means clustering of protein trajectories was completed using the hkmeans function in the *factoextra* package (33). Cluster size was fixed at six for *X. laevis* and *D. melanogaster*. For *C. robusta*, clustering was initially done with 10 clusters to ensure that the maternally deposited proteins were separated from other protein classes. Proteins in this cluster were reassigned after reclustering with five clusters. Functional enrichment analysis was complete d with the gprofiler2 package (34).

## Results and Discussion

### RTS Filter Improves TMTc Duty Cycle by Intelligently Selecting Precursors for Long-Transient Orbitrap Scans

RTS increases sensitivity of SPS-MS3 methods by informed selection of precursor peaks for quantification (15, 35). SPS-MS3 methods have longer duty cycles than standard MS2-based reporter ion quantification methods owing to the extra MS3 scan needed per PSM. RTS reduces the extra time needed for MS3 scans by limiting MS3 scans to precursors that have been matched to a peptide. We reasoned that the same time-saving strategy could be applied to complementary ion quantification (TMTc or TMTproC) scans, which also use considerable IIT relative to MS2-based reporter ion quantification.

**Fig. 1:**
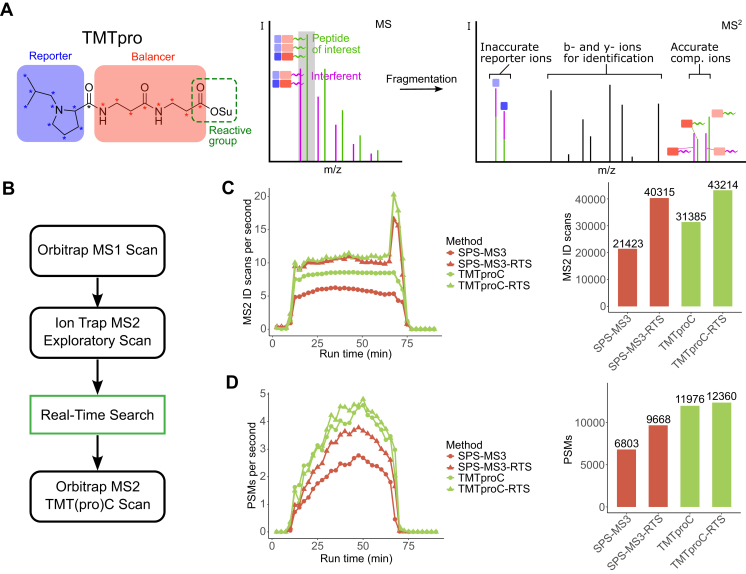
**Principle of TMTproC and comparison of duty cycle between TMTproC and TMTproC-RTS.** TMT-labeled yeast TKO standard was used to evaluate the duty cycle effects of RTS on TMT(pro)C analyses. *A*, principle of complementary ion quantification. When isolating a precursor for fragmentation, in addition to the peptide of interest (*green*), other peptides with similar *m/z* ratio (interferents, *purple*) will be coisolated (*gray box*). If MS2 reporter ions are used for quantification, the interfering peptides lead to a distortion of the measured ratios. However, because the masses of complementary ions are peptide dependent, they can be used for interference-free accurate MS2 quantification. *B*, overview of the TMT(pro)C-RTS workflow. Precursors from MS1 spectra are isolated and fragmented, and an exploratory MS2 scan is taken in the ion trap. Spectra are searched in real time against a FASTA file. If a peptide match is found, a second MS2 scan is collected in the high-resolution Orbitrap with longer ion injection time. *C*, the TMT-labeled yeast TKO standard was analyzed with TMTproC and SPS-MS3 with and without an RTS filter. The duty cycle of each method is plotted throughout the method gradient. The bar plot shows the total identification scans (Orbitrap scans for TMTproC and ion trap scans for all other methods) across the entire run. RTS saves instrument time that can be used on identification scans. *D*, from the same experiment as in *B*, the PSMs for TMTproC and SPS-MS3 with and without RTS throughout the method gradient. The bar plot shows the total PSMs across the entire run. TMTproC-RTS had the most PSMs of the four methods. PSM, peptide spectral match; RTS, real-time search; TKO, triple knock-out standard; TMT, tandem mass tag.

To this end, we selected precursors for isolation from MS1 scans in a data-dependent manner and analyzed them in the ion trap. We sought to maximize scans that could be used for identification by using the fastest ion trap scan speed (turbo), limiting maximum IITs to 13 ms, using HCD for peptide fragmentation, and limiting the scan range to 200 to 1400 *m/z*. These “exploratory” scans were then searched against an appropriate FASTA file, and positive hits were reisolated, fragmented, and analyzed by the Orbitrap for complementary ion quantification (Fig. 1*A*).

We evaluated TMTc-RTS, TMTc, SPS-MS3-RTS, and SPS-MS3 on 1 μg shots of the *Saccharomyces cerevisiae* (yeast) triple knock-out standard labeled with TMT 10-plex (36) with 80-min unfractionated runs. Any peptide that could be matched to a yeast protein and passed quality filters (see *Experimental procedures* section) was used to trigger a long-transient TMTc scan or MS3 scan, depending on the method used.

Compared with TMTc, TMTc-RTS collected 37% more MS2 scans that could lead to the identification of a peptide (Fig. 1*B*). The increase in MS2 ID scans with RTS led to a 3% increase in PSMs for TMTc (Fig. 1*C*). The relatively small gain in PSMs compared with the increase in number of scans can be attributed to the low resolution and injection times of the ion trap scans.

TMTc methods had more PSMs than SPS-MS3 methods regardless of RTS use. The number of MS2 ID scans was comparable between SPS-MS3-RTS and TMTc-RTS (40,315 and 43,214, respectively), whereas the number of PSMs favored TMTc-RTS by a larger relative margin (9668 and 12,360 PSMs, respectively). Here, the number of MS2 scans that pass the RTS filter and trigger a quantification scan explained the difference in PSMs between the two methods (11,365 for SPS-MS3-RTS and 13,819 for TMTc-RTS). The difference is likely because of the different fragmentation techniques used for each method. SPS-MS3-RTS uses CID fragmentation because ion handling is more efficient and the MS2 and MS3 spectra need to use the same fragmentation technique and normalized collision energy for proper setting of the SPS notches. In contrast, TMTc-RTS uses HCD fragmentation for the exploratory MS2 scans, which tends to form a greater diversity of b- and y-ions for improved peptide identification rates.

The relative gain in MS2 ID scans and PSMs using RTS were smaller for TMTc (37% and 3%, respectively) than for the SPS-MS3 method (88% and 42%, respectively). In both methods, RTS sensitivity improvements come from the ability to spend less analysis time on a precursor if it will not lead to a positive identification and quantification. However, TMTproC-RTS adds an extra ion trap scan for every possible precursor, whereas the SPS-MS3-RTS method does not collect any additional scans relative to SPS-MS3. Furthermore, TMTproC-RTS trades high-resolution Orbitrap ID scans for low-resolution ion trap ID scans, which likely have lower success rates. Nevertheless, we reasoned that TMTproC-RTS might be beneficial for targeting a subset of the proteome, or for quantification of prefractionated samples, where the fraction of successful identification decreases.

### The Improvement in Sensitivity Using TMTproC-RTS Is a Function of Target Protein List Size

Next, we explored to what extent TMTproC-RTS outperforms standard TMTproC when selecting a subsection of the proteome as target. We expected that the increase in PSMs with TMTproC-RTS relative to TMTproC would be a function of the success rate of MS2 scans. The more futile TMTproC scans performed during a run, the more benefit that an RTS step would confer. One common experiment type with lower “success” rates would be those where researchers are only interested in the behavior of a subset of the proteins in a complex sample. PSMs that map to nontarget proteins would be considered futile in these experiments. We implemented a semitargeted TMTproC-RTS method by modifying the FASTA file used for RTS. We added a text tag to the header of proteins not in the target list. The same tag was added to the RTS node of the method editor as an exclusion criterion so that PSMs that mapped to those proteins would not trigger a TMTproC scan.

**Fig. 2:**
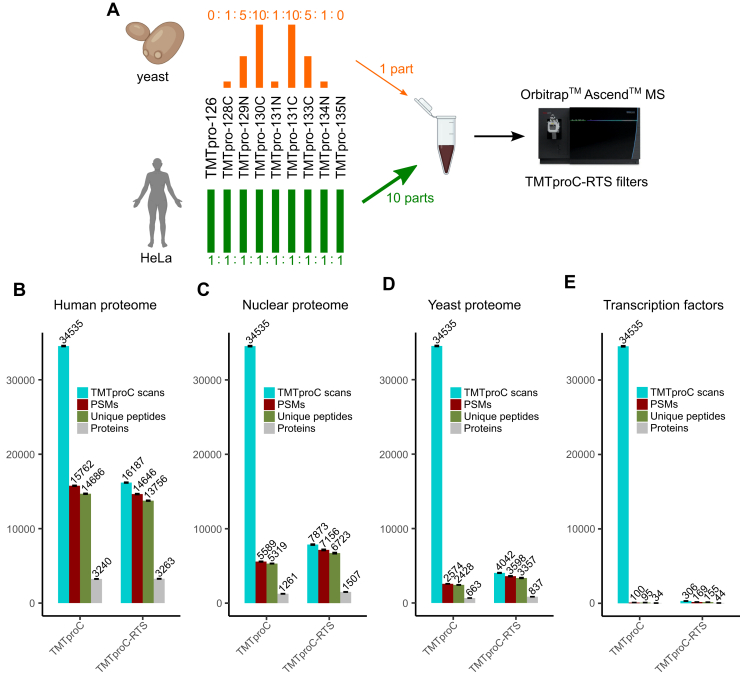
**TMTproC-RTS peptide and protein identifications as a function of the target protein list.***A*, yeast peptides were labeled with TMTpro in ratios of 0:1:5:10:1:10:5:1:0 across the nine complementary ion channels. Similarly, HeLa peptides were labeled in ratios of 1:1 across the nine channels. Yeast and HeLa peptides were mixed at ratios of 1 part yeast to 10 parts HeLa and analyzed with TMTproC(-RTS) in triplicate. *B*–*E*, in four different experiments, the RTS filter was set to trigger on a different set of proteins: the human proteome, nuclear proteome, yeast proteome, and yeast/human transcription factors. TMTproC-RTS improved protein identifications by 0%, 20%, 26%, and 29% for these target lists, respectively. Values plotted are the mean of three replicates. Error bars represent the standard deviation of replicates. RTS, real-time search; TMT, tandem mass tag.

We evaluated the semitargeted TMTproC-RTS method on a mixed sample of HeLa and yeast peptides. Yeast peptides were labeled in ratios of 0:1:5:10:1:10:5:1:0 across the nine complementary ion channels. HeLa peptides were labeled in ratios of 1:1. Yeast and HeLa peptides were mixed in ratios of 1 part yeast to 10 parts HeLa before analysis (Fig. 2*A*). We selected target protein lists across a range of sizes. These include human proteins (86% of peptides in TMTproC runs), nuclear proteins (29% of peptides in TMTproC runs), yeast proteins (13% of peptides in TMTproC runs), and transcription factors (TFs) (0.5% of peptides in TMTproC runs).

As expected, the increase in protein identifications was more pronounced for smaller target protein lists. TMTproC-RTS improved protein identifications by 0%, 20%, 26%, and 29% for the human, nuclear, yeast, and TF target lists, respectively (Fig. 2*B*). The ratio of triggered TMTproC scans to successful peptide identifications was relatively constant across the series, with success rates of 90%, 91%, and 89% for the human proteome, nuclear, and yeast target lists, respectively. However, the success rate dropped to 55% using a TF target list. With very small target lists, it is possible that gas phase fractionation using an ion mobility device or limited MS1 ranges could increase the number of peptide identifications. We also explored the use of the Close-out feature, which did not increase the number of identified proteins (Supplemental Fig. S5). These results show that TMTproC-RTS improves peptide identifications more dramatically when targeting a subset of proteins in a complex sample.

### Compared With TMTproC, TMTproC-RTS Shows Favorable Trade-Offs Between Sensitivity and Data Quality

A trade-off exists in bottom–up quantitative proteomics experiments between sensitivity and measurement accuracy and precision. Longer IITs and longer transients with higher resolution for the quantification of each precursor come at the cost of slower spectral acquisition rates and therefore fewer quantified precursors. TMTproC uses a comparatively high maximum MS2 IIT with a matching transient length of 90 ms (45k resolving power on an Ascend Tribrid MS). A higher max IIT affords more ions and enables the quantification of lower abundance precursors.

**Fig. 3:**
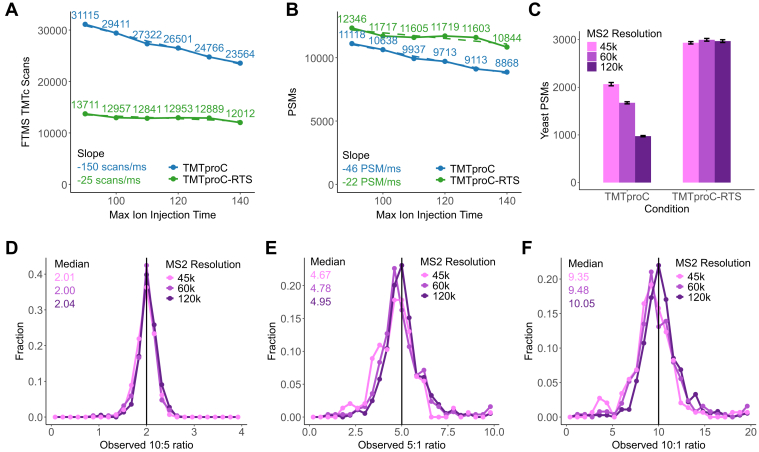
**The tradeoff between quantification precision and sensitivity is more favorable with TMTproC-RTS *versus* TMTproC.***A* and *B*, the yeast TKO standard was successively analyzed with TMTproC(-RTS) with maximum MS2 ion injection times ranging from 90 to 140 ms. The number of TMTc scans and PSMs for each method is plotted as a function of maximum ion injection time. TMTproC-RTS loses fewer scans and PSMs at higher injection times. *C*, the HeLa–yeast interference sample from Figure 2 was analyzed with three different MS2 Orbitrap resolutions (45k, 60k, and 120k) with and without RTS. For the TMTproC-RTS analyses, the RTS filter was set to target peptides originating from yeast proteins. Error bars represent the standard deviation of triplicate measurements. The increased transient lengths had no effect on TMTproC-RTS sensitivity. *D*–*F*, from the TMTproC-RTS runs in *C*, the complement ion ratios for three channel combinations were extracted for unique yeast PSMs that were seen in all three conditions with a total signal to Fourier transform noise ratio above 200. The higher resolution improves the precision and accuracy of TMTproC measurements. PSM, peptide spectral match; RTS, real-time search; TKO, triple knock-out; TMT, tandem mass tag.

We tested the effect of max IIT over the 90 to 140 ms range on repeated shots of 1 μg of yeast triple knock-out standard with an 80-min gradient. A 45k resolution Orbitrap scan has about 90 ms of overhead, so a lower max IIT is unnecessary. We found that using TMTc, each additional millisecond of max IIT led to 150 fewer MS2 scans throughout the run and 46 fewer PSMs (total drop of 24% and 20% going from 90 to 140 ms, respectively). In contrast, while using TMTc-RTS, each millisecond of max IIT led to 25 fewer TMTc scans and 22 fewer PSMs (total drop of 12% and 12% going from 90 to 140 ms, respectively) (Fig. 3, *A* and *B*).

We next tested how Orbitrap resolution would affect the sensitivity-accuracy trade-off. The ability of TMTproC to distinguish between signal from the peptide of interest and interfering peptides is a function of the scan resolution. Higher resolutions are expected to improve accuracy and precision. Here, we analyzed repeated shots of the TMTpro-labeled HeLa–yeast interference model system at 45k, 60k, and 120k MS2 resolutions with TMTproC and TMTproC-RTS. In RTS runs, the RTS filter was set to isolate peptides stemming from yeast proteins. Max IITs were set at 91, 123, and 251 ms, respectively.

In TMTproC runs, quantified yeast PSMs were similar between 45k and 60k resolution but dropped by half between 60k and 120k resolution. TMTproC-RTS quantified a similar number of yeast PSMs at all resolution settings (Fig. 3*C*).

In the TMTproC runs, about 50 min of the 80-min gradient is spent collecting the ∼35,000 TMTproC scans at 45k resolution. It would take about 145 min to keep the same number of scans at 120k resolution, which causes a large drop in sensitivity. In 45k resolution TMTproC-RTS runs, the ∼2000 TMTproC scans only account for about 3 min of the gradient. At 120k resolution, this is increased to 10 min, which can be easily accomplished by omitting exploratory scans of the lowest abundant precursors that were likely not quantified at 45k resolution.

As hypothesized, a higher Orbitrap resolution improved quantification of yeast PSMs. The median 10:5 ratio of yeast PSMs for the TMTproC-RTS runs were between 2.00 and 2.04 for all runs, accurately retrieving the correct ratio. For the 5:1 ratios calculated from the outer portion of the complement ion cluster, the 120k Orbitrap resolution improved the median ratio from 4.67 to 4.95 (Student’s *t* test, *p* = 0.0009). Similarly, the median 10:1 ratio improved from 9.35 to 10.05 when Orbitrap resolutions were increased from 45k to 120k (Student’s *t* test, *p* = 0.0001) (Fig. 3, *D*–*F*). These results demonstrate that TMTproC-RTS can be used to improve peptide quantification without loss of sensitivity when a subset of the proteome is targeted.

### TMTproC-RTS Improves Sensitivity of Proteomic Time Courses of Embryogenesis in *D. melanogaster*, *C. robusta*, and *X. laevis*

Understanding how a single cell transforms into a complex organism is one of the central goals of developmental biology. Efforts to study this process have been aided by various omics measurements across development. We sought to leverage the sensitivity and accuracy of TMTproC-RTS to generate valuable resources for the respective developmental biology communities. To this end, we analyzed new time courses of proteome dynamics for the embryonic development of three model organisms, *D. melanogaster* (fly), *C. robusta* (sea squirt), and *X. laevis* (frog).

**Fig. 4:**
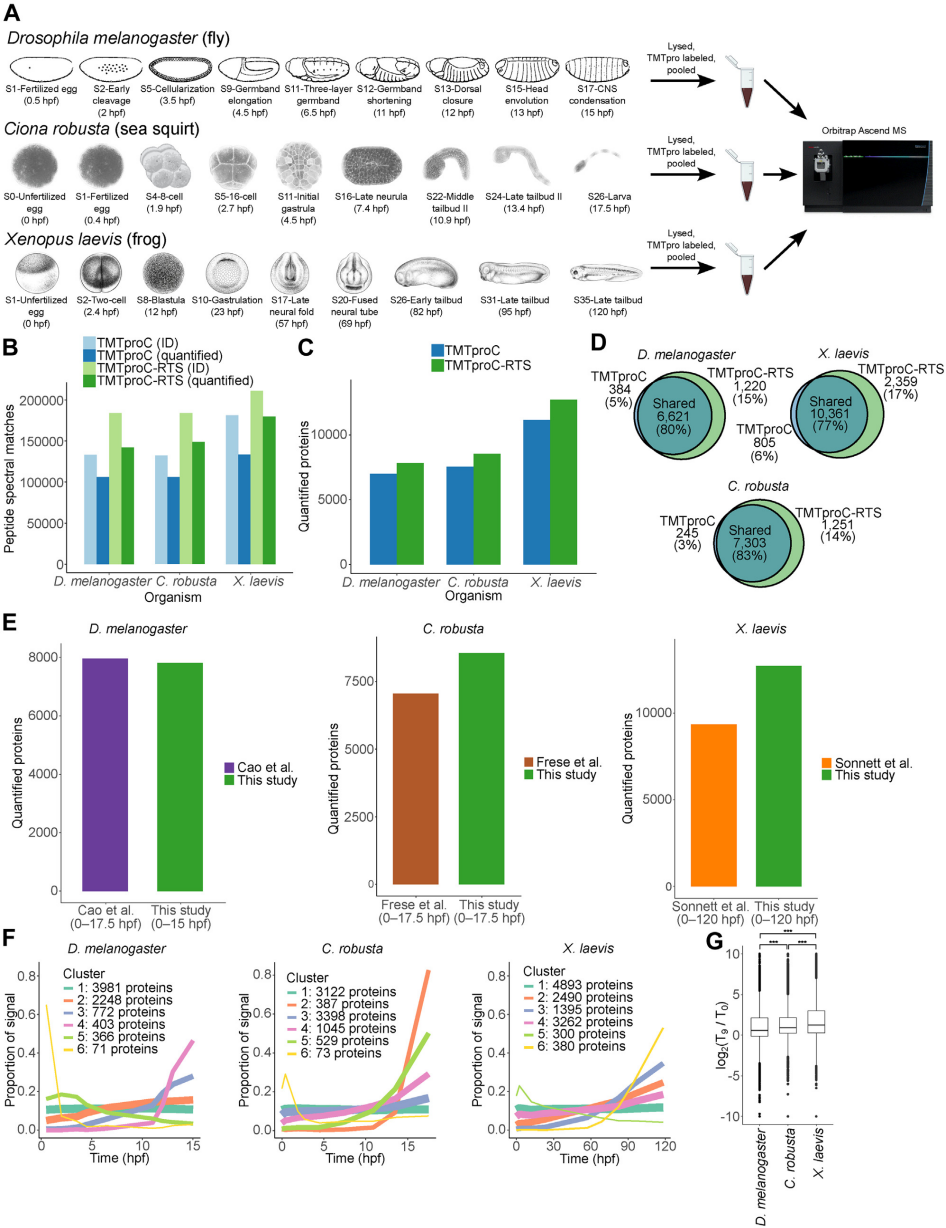
**Application of TMTproC-RTS to protein dynamics in fly, frog, and sea squirt development advances resources available for those model systems.***A*, *Drosophila melanogaster* (fly), *Ciona robusta* (sea squirt), and *Xenopus laevis* (frog) embryos were collected at the stages shown (23, 26, 41, 42). Hours postfertilization (hpf) values are approximate for each stage. *B*, each time course was fractionated into 24 samples, and each fraction was analyzed with TMTproC and TMTproC-RTS. The total number of PSMs that were identified and quantified are shown for each method and organism. TMTproC-RTS improved PSMs by 15 to 40% and peptide quantifications by 35 to 40% for each organism compared with TMTproC. *C*, TMTproC quantified 7005, 7548, and 11,166 proteins in fly, sea squirt, and frog, respectively, whereas TMTproC-RTS quantified 7841, 8554, and 12,720 proteins, respectively (12%, 13%, and 14% increase). *D*, overlap between the proteins quantified by TMTproC and TMTproC-RTS. *E*, number of quantified proteins in similar time courses of other studies. The Cao *et al*. time course includes later developmental stages when more proteins are expressed, which likely explains the relatively large number of proteins. *F*, hierarchical k-mean clusters of all proteins quantified in TMTproC-RTS datasets. *G*, Log2-fold ratios between the first and last time point were calculated for each protein. Boxplots display the 25th, median, and 75th percentile, with whiskers extending 1.5 times the interquartile range. ∗∗∗*p* < 0.001, Wilcoxon rank-sum test. About 7% of *D. melanogaster* proteins decreased by more than twofold across the time course compared with 4% and 3% for *C. robusta* and *X. laevis*, respectively (Fisher’s exact test, *p* = 6e-14 and <2e-16). PSM, peptide spectral match; RTS, real-time search; TMT, tandem mass tag.

Each set of embryos was visually staged, and samples collected at nine developmental milestones (Fig. 4*A*). TMTpro-labeled peptides from each time course were fractionated into sets of 24 before analysis with TMTproC and TMTproC-RTS. TMTproC-RTS improved PSM quantifications by 36%, 40%, and 35% compared with TMTproC for *D. melanogaster*, *C. robusta*, and *X. laevis*, respectively (Fig. 4*B*). PSM gains translated into an increase in protein quantifications of 12, 13, and 14% for each organism, respectively (Fig. 4, *C* and *D*). In addition, the increases in protein quantifications for signaling proteins and TFs were 8%, 15%, and 26%. These two classes of proteins are of particular interest to developmental biologists and are difficult to quantify using MS owing to their low abundance.

We also compare the number of quantified proteins with previous studies that collected similar time courses in each organism (Fig. 4, *E*–*G*, Supplemental Fig. S3). Sonnett *et al.* (17) collected a 10-sample TMT-MS3 time course throughout the development of *X. laevis*. TMTproC-RTS quantified 36% more proteins than this study, with added sensitivity from both TMTproC and RTS. Cao *et al.* (37) collected a five-sample TMTc time course throughout the development of *D. melanogaster*. TMTproC-RTS quantified 1% fewer proteins than this study. The decrease in the number of quantified proteins compared with Cao *et al.* is likely because the last time point was ∼2.5 h later in development, which results in more differentiated tissues and many novelly expressed proteins that were not accessible in this study. Proteins that were uniquely detected by Cao *et al.* and not in the current study show a strong bias in their abundance profile toward the final time point (Supplemental Fig. S4). If proteins with more than 80% of their signal in the final time point are excluded, TMTproC-RTS quantified 14% more proteins than Cao *et al.* TMTproC-RTS quantified 21% more proteins than an eight-sample TMTproC time course of *C. robusta* embryogenesis collected by Frese *et al.* (38).

The proteomes of the three organisms show broadly similar behaviors and were highly reproducible (Supplemental Figs. S1 and S2). The largest cluster of proteins (hierarchical k-mean clustering) in each organism was constant across embryogenesis (Fig. 4*F*). Functional enrichment analysis of this cluster (g:Profiler (34)) indicates that these are “housekeeping” proteins associated with the mitochondria, ribosomes, and the cytoskeleton. Similarly, the second largest cluster in each organism increased slightly over embryogenesis, indicating that the majority of protein levels are static throughout development. The most dynamic clusters in each organism are composed of a few hundred proteins that are likely of highest interest to developmental biologists.

Proteins whose abundance decreased across embryogenesis were more prevalent in *D. melanogaster* than the other two organisms. More than 6% of *D. melanogaster* proteins are found in the two clusters with a decreasing profile (Fig. 4*F*, yellow and light green clusters). In contrast, in *C. robusta*, less than 1% of proteins fall into the decreasing cluster and 2% in *X. laevis* (yellow and light green clusters, respectively). Similarly, the proportion of proteins with more than a twofold decrease in abundance from the first to the last time point was 7%, 4%, and 2% in *D. melanogaster*, *C. robusta*, and *X. laevis*, respectively (Fig. 4*G*, Fisher’s exact test, *p* = 6e-14 and <2e-16). One possible cause is that degradation of maternally deposited proteins plays a larger role in *D. melanogaster* embryogenesis.

**Fig. 5:**
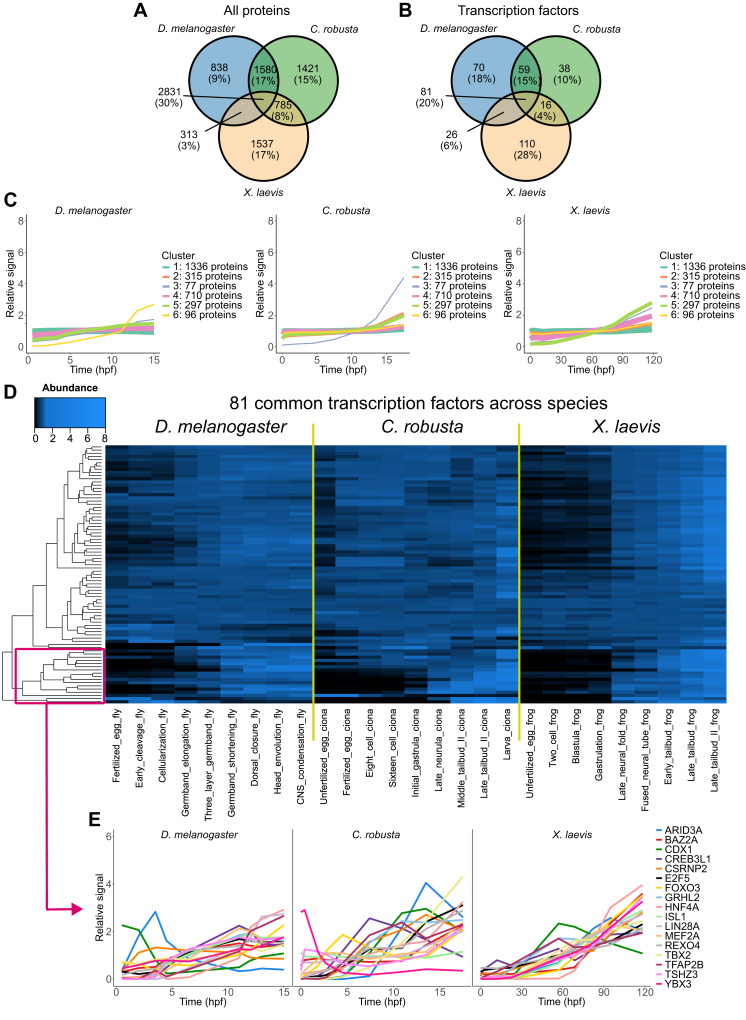
**Comparison of protein dynamics across species.***A*, proteins from the three organisms were mapped to the human proteome using OrthoFinder (32). Venn diagram shows the overlap between orthologs that were detected and quantified in each dataset. *B*, As in *A* after filtering for orthogroups containing known human transcription factors. *C*, protein dynamics were coclustered using hierarchical k-means clustering for the proteins that were quantified in all three organisms. Proteins that were stable across development clustered together, whereas more dynamic proteins tended to be species specific. *D*, heat map showing the abundance profiles of the transcription factors that were quantified in all three organisms. *E*, abundance profiles of 17 transcription factors from the clustering in *D*.

Proteins from the three organisms were mapped to the human proteome using OrthoFinder for cross-species comparison (32). This analysis identified 2831 orthogroups that contained proteins detected in all three organisms, accounting for approximately 30% of the common orthogroups among the species (Fig. 5, *A* and *B*). *D. melanogaster* and *X. laevis* are evolutionarily separated by 708 million years and shared the fewest unique orthologous proteins (∼3%), whereas the closer *C. robusta* and *X. laevis* (588 million years) shared ∼8% of their orthologous proteins uniquely between them (Fig. 5, *A* and *B*) (39). Notably, 81 of the orthogroups included human TFs that were detected in all three organisms, representing 20% of all common orthologs identified.

Next, using the set of 2831 common orthologs, we performed hierarchical k-means clustering to cocluster these proteins based on their abundance profiles during embryogenesis (Fig. 5*C*). The largest cluster of common proteins, represented by the dark green line in Figure 5*C*, did not change abundance across any of three organisms. However, dynamic clusters tended to behave differently in each organism. For example, the 96-protein cluster (yellow in Fig. 5*C*) was the most dynamic in *D. melanogaster*, increasing by more than fourfold, whereas its increase was more subtle in *C. robusta* and *X. laevis*.

Next, we focused on protein dynamics of 81 orthologous TFs, critical regulators of cell fate specification and identity, that were detected in all three organisms (Fig. 5*D*). While the behavior of some proteins was similar across organisms (*e.g.*, HNF4A, LIN28A, and TFAP2B all increase by similar magnitudes), others have diverged (Fig. 5*E*). For example, the CDX1 orthologs peak in abundance during the tailbud II stage in *C. robusta*, prior to tail formation in the *X. laevis*, and in the fertilized egg in *D. melanogaster*. Other interesting examples include YBX3 whose ortholog is rapidly degraded in the *C. robusta* early embryogenesis, and ARID3A, whose maximum abundance varies between the organisms (Fig. 5*E*). Thus, we provide valuable resources for the developmental community that will help researchers generate hypotheses and better understand embryogenesis (Supplemental Table S1).

## Conclusion

This study introduces a method combining complementary ion quantification with RTS (TMTproC-RTS). TMTproC-RTS improves sensitivity while maintaining accuracy and precision in quantitative proteomics experiments at the MS2 level. While sensitivity gains were modest for whole proteome, single-shot analyses, we found that TMTproC-RTS increases protein identifications by up to 29%, depending on the RTS target list's proteome proportion. In addition, the sensitivity of prefractionated proteomes is markedly improved by TMTproC-RTS. For example, prefractionated TMTproC-RTS analyses of *D. melanogaster*, *C. robusta*, and *X. laevis* embryos quantified 12%, 13%, and 14% more proteins than TMTproC.

We provide proof of principle for targeting specific protein lists without prior knowledge of their abundance or peptide properties. This method is promising for TFs, signaling molecules, and other defined proteome subsets. However, the method is currently limited by the number of observable precursors in the MS1 spectrum. In principle, this limitation can be overcome by employing data-independent acquisition (DIA) to identify precursors of interest in real time for quantification. Although DIA-RTS is currently inaccessible, we believe it is a matter of time before this becomes available. Such a TMTproC-DIA-RTS would be particularly beneficial for instruments that rely on fast scanning DIA methods, which have extremely fast duty cycles for identification but lack MS3 capabilities. Implementing TMTproC-DIA-RTS on these instruments should be straightforward once DIA-RTS becomes available.

We demonstrate the power of TMTproC-RTS by generating comprehensive resources for protein dynamics during the embryogenesis of *D. melanogaster*, *C. robusta*, and *X. laevis*. These resources will be valuable for the respective developmental biology communities. In each study, we increased the number of quantified proteins while simultaneously improving measurement accuracy and precision. These resources are particularly valuable for evolutionary comparisons of proteomes in developmental progression. Proteomic studies, like those by Frese *et al.*, show great promise and reveal insights not apparent or even contrary to mRNA-based studies.

Thus, TMTproC-RTS advances current proteomics technology, provides valuable resources for developmental biology, and demonstrates potential for future integration with next-generation instrumentation to further improve proteomic analyses.

## Data availability

The MS proteomics data have been deposited to the ProteomeXchange Consortium *via* the PRIDE (40) partner repository with the dataset identifier PXD055647.

## Supplemental Data

This article contains supplemental data.

## Conflict of interest

G. M., W. B., J. C., J. H., R. M., and V. Z. are employees of ThermoFisher Scientific. Thermo Fisher provides limited support to M. W. laboratory under a collaborative research agreement with Princeton University. All other authors declare that they have no conflicts of interest with the contents of this article.

## Supplemental Data

Supplemental Table S1

Supplemental Caption
