# Supplementary material for: Sensitive and Accurate Proteome Profiling of Embryogenesis Using Real-Time Search and TMTproC Quantification
Source: Mol Cell Proteomics. 2024 Dec 24;24(2):100899. doi: 10.1016/j.mcpro.2024.100899 (PMC11815649; doi:10.1016/j.mcpro.2024.100899)
Supplement: Supplemental Caption [file mmc2.docx]

**Figure S1. Technical reproducibility of proteomic development time courses.** Pearson correlation coefficients were calculated between replicate measurements of *D. melanogaster* (**A**), *C. robusta* (**B**), and *X. laevis* (**C**) with TMTproC and TMTproC-RTS for all time points. All correlation coefficients were above 0.96.

**Figure S2. Coefficient of variation measurements.** Coefficient of variation (standard deviation divided by the mean, CV) distributions were calculated for replicate measurements for each proteomic time course of embryonic development for *D. melanogaster* (**A**), *C. robusta* (**B**), and *X. laevis* (**C**). Boxplots indicate median, 25^th^, and 75^th^ percentiles. The median CV for each organism was 0.028, 0.031, 0.030 for fly, sea squirt, and frog, respectively.

**Figure S3. Comparison with previous proteomic time courses of embryonic development.** The cosine similarity between this study and other comparable developmental time series in *D. melanogaster* (**A**), *C. robusta* (**B**) and *X. laevis* (**C**) was calculated for each gene that was detected in both studies. For *D. melanogaster*, there were four overlapping time points (fertilized egg, early cleavage, germband elongation, and germband shortening) with Cao *et al*. For *C. robusta*, there were eight overlapping time points (all but the eight-cell stage) with Frese *et al*. For *X. laevis*, there were 9 overlapping time points (additional stage 12 was removed) with Sonnett *et al*. The data sets were in strong agreement with previous time courses.

**Figure S4. Abundance of *D. melanogaster* proteins unique to Cao *et al*. and this study.** The abundances of proteins unique to Cao *et al*. (**A**) are highly skewed towards the 17.5-hour post-fertilization time point. The Cao *et al*. time course collected the final time point ~2.5 hours later in development, which resulted in more differentiated tissues and many novelly-expressed proteins that were not accessible in this study. In contrast, proteins unique to this study (**B**) were not enriched in any time point. Boxplots represent 25^th^, median, and 75^th^ percentile.

**Figure S5. Effect of the Close-out feature on TMTproC-RTS.** HeLa peptides were labeled with TMTpro0 and analyzed with TMTproC-RTS with and without the Close-out feature enabled. The Close-out feature suppresses RTS triggers if N peptides have already been identified from the current protein match. Here we tested settings of either 2 or 3 peptides for Close-out. The Close-out feature decreased the number of peptide spectral matches and unique peptides and had no effect on the number of identified proteins. Values plotted are the mean of three replicates. Error bars represent the standard deviation of replicates.

**Figure S6. Functional enrichment analysis of constant cluster in developmental time course.** Hierarchical k-means clustering of protein trajectories was completed for each organism. The largest cluster for each organism had a constant abundance throughout development (cluster number 1 in Figure 4F). We performed function enrichment analysis on that cluster using g:Profiler. The top five categories with a *p*-value (Benjamini-Hochberg false discovery rate corrected) of less than 0.01, an odds ratio of greater than 1.5, and were “highlighted” by g:Profiler algorithm were selected for each organism. Odds ratios were calculated as the ratio of the odds that a gene was found in the constant cluster given that it was a member of the set to the odds that a gene was found in the constant cluster given that it was not a member of the set.
