# Supplementary figures and images for: Sensitive and Accurate Proteome Profiling of Embryogenesis Using Real-Time Search and TMTproC Quantification
Source: Mol Cell Proteomics. 2024 Dec 24;24(2):100899. doi: 10.1016/j.mcpro.2024.100899 (PMC11815649; doi:10.1016/j.mcpro.2024.100899)

**A**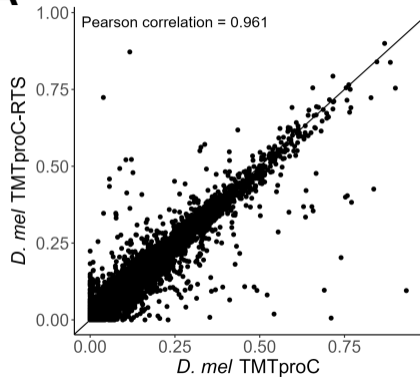**B**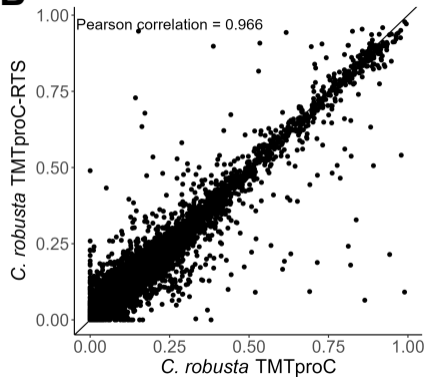**C**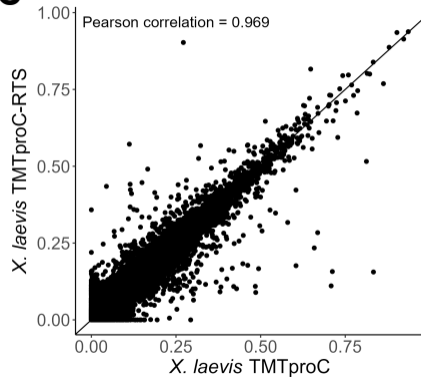

Supplement: Supplemental Figure S1 [file mmc3.pdf]

**A**

Median CV = 0.028

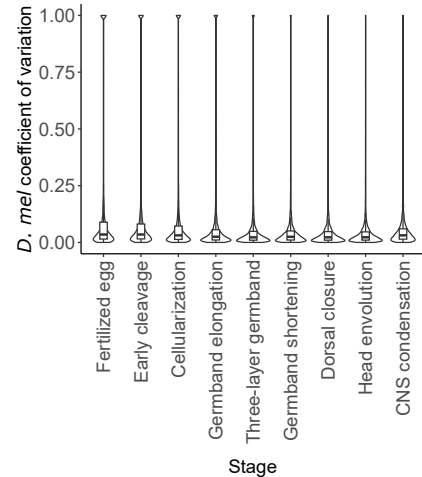**B**

Median CV = 0.031

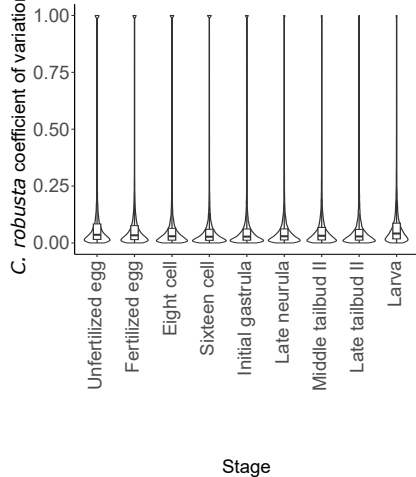**C**

Median CV = 0.030

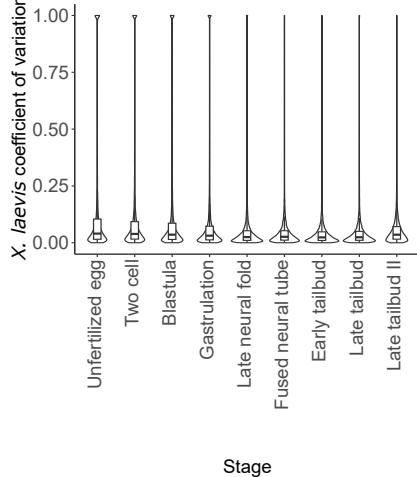

Supplement: Supplemental Figure S2 [file mmc4.pdf]

**A***D. melanogaster*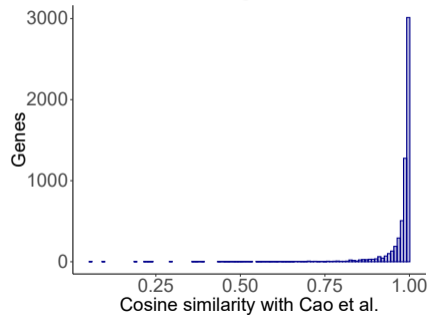**B***C. robusta*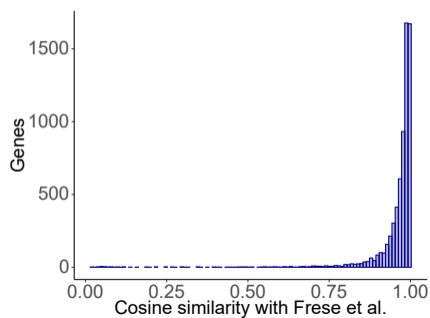**C***X. laevis*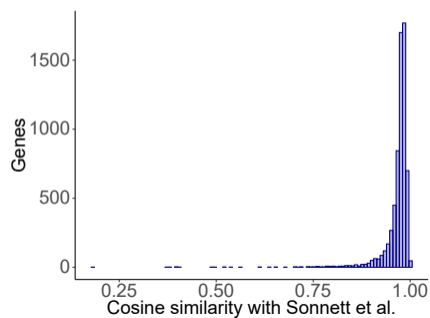

Supplement: Supplemental Figure S3 [file mmc5.pdf]

**A**

Proteins unique to Cao et al.

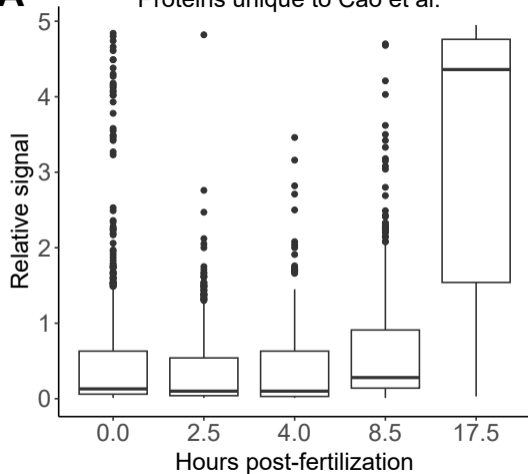**B**

Proteins unique to this study

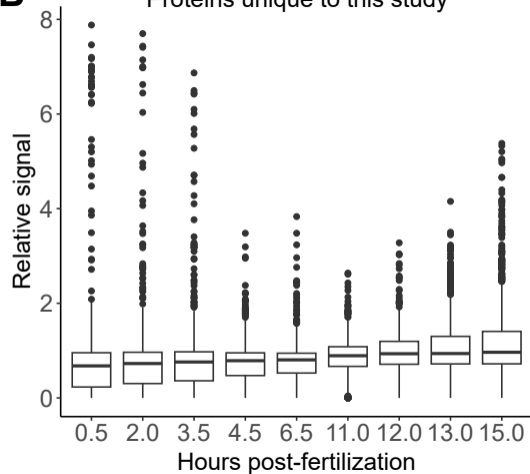

Supplement: Supplemental Figure S4 [file mmc6.pdf]

# Method

- TMTproC-RTS
- TMTproC-RTS Close-out (3)
- TMTproC-RTS Close-out (2)

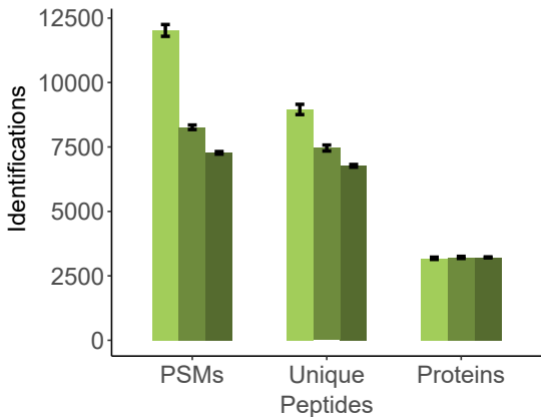

Supplement: Supplemental Figure S5 [file mmc7.pdf]
