# Supplementary material for: Sensitive and Accurate Proteome Profiling of Embryogenesis Using Real-Time Search and TMTproC Quantification
Source: Mol Cell Proteomics. 2024 Dec 24;24(2):100899. doi: 10.1016/j.mcpro.2024.100899 (PMC11815649; doi:10.1016/j.mcpro.2024.100899)
Supplement: Supplemental Figure S6 [file mmc8.pdf]

D. melanogaster

Term Name	Odds Ratio	-log10(*p*-value)
Mitochondrion	4.71	109.5
Structural constituent of the ribosome	17.11	55.8
Cell cycle	2.59	34.4
RNA binding	2.06	17.7
Supramolecular complex	1.81	7.2

C. robusta

Term Name	Odds Ratio	-log10(*p*-value)
Microtubule organization	2.85	23.1
Mitochondrial respiratory chain complex assembly	9.02	22.5
ncRNA processing	2.48	11.2
Spliceosomal complex	3.37	11.2
Protein-macromolecule adaptor activity	1.55	4.6

X. laevis

Term Name	Odds Ratio	-log10(*p*-value)
Mitochondrion organization	4.30	44.4
tRNA metabolic process	6.35	32.3
Modification-dependent macromolecule catabolic process	2.53	19.2
Mitotic spindle	2.55	6.3
Ribosome biogenesis	1.69	2.6
